# Prevalence of Intestinal Protozoa among Saudi Patients with Chronic Renal Failure: A Case-Control Study

**DOI:** 10.1155/2015/563478

**Published:** 2015-09-28

**Authors:** Yousry A. Hawash, Laila Sh. Dorgham, El-Amir M. Amir, Osama F. Sharaf

**Affiliations:** ^1^Department of Medical Laboratory Science, College of Applied Medical Sciences, Taif University, Taif 21944, Saudi Arabia; ^2^Department of Medical Parasitology, National Liver Institute, Menoufia University, Shebin El-Koom, Menoufia 23513, Egypt; ^3^Department of Community Medicine, National Liver Institute, Menoufia University, Shebin El-Koom, Menoufia 23513, Egypt; ^4^Parasitology Department, Rabigh Medical College, King Abdulaziz University, Jeddah 21589, Saudi Arabia

## Abstract

It has been hypothesized that chronic renal failure (CRF) predisposes patients to infection with intestinal protozoa. We tested this hypothesis with a matched case-control study to determine the prevalence of these protozoa and their diarrhea associated symptoms among 50 patients with CRF (cases) from Taif, western Saudi Arabia. Fifty diarrheal patients without CRF were recruited in the study as controls. Participants were interviewed by a structured questionnaire and stool samples were collected. Samples were thoroughly examined with microscopy and three coproantigens detection kits. Enteric protozoa were detected in 21 cases and 14 controls. *Blastocystis* spp. were the most predominant parasite (16% in cases versus 8% in controls), followed by *Giardia duodenalis* (10% in cases versus 12% in controls) and *Cryptosporidium* spp. (10% in cases versus 6% in controls). *Cyclospora cayetanensis* was identified in two cases, while *Entamoeba histolytica* was described in one case and one control. Intestinal parasitism was positively associated with the male gender, urban residence, and travel history. Clinical symptoms of nausea/vomiting and abdominal pain were significantly varied between the parasitized cases and controls (*P* value ≤ 0.05). Given the results, we recommend screening all diarrheal feces for intestinal protozoa in the study's population, particularly those with CRF.

## 1. Introduction

Enteric protozoa are a diverse group of unicellular microparasites inhabiting the intestinal tract of high vertebrate hosts including humans [1]. Infections usually occur through ingestion of cysts/oocysts contaminating raw food or drinking water [2, 3]. Diarrhea is relatively a frequent symptom for protozoan infections but asymptomatic colonization is also common [1]. Attributing diarrhea to an exact parasite identified in a patient's feces is not certain for all protozoa. While a number of intestinal protozoa such as* Giardia duodenalis*,* Entamoeba histolytica*,* Cryptosporidium* spp.,* Cyclospora cayetanensis*,* Cystoisospora belli*, and Microsporidia spp. have been certainly recognized to cause diarrhea in humans [4], others like* Entamoeba coli* and* Entamoeba dispar* almost certainly have not [5]. In addition, enteric protozoa like* Blastocystis* spp. and* Dientamoeba fragilis* have been recently identified in patients with diarrhea, but their causal role is still uncertain [6]. Regardless of the underlying protozoan parasite, diarrhea is usually mild and self-limited in healthy immunocompetent persons. Nevertheless, severe and protracted diarrhea has been reported in immunosuppressed patients [7].

By definition, patient with chronic renal failure (CRF) is a patient with end stage kidney disease causing marked decline in the glomerular filtration rate as well as uremia and requires kidney replacement or scheduled dialysis to survive [8]. It has been hypothesized that patients, at this stage, are more susceptible to infectious diarrhea than the normal population, secondary to immunosuppression [9, 10]. This infectious diarrhea has been found associated with a number of intestinal viruses, bacteria, and protozoa [11]. In Saudi Arabia, high prevalence of enteric protozoa-related diarrhea has been reported [12] and the number of patients with CRF has been found rising [13]. Nevertheless, the frequency of these protozoa among patients with CRF has been poorly studied. In a case-control study, we tested the above hypothesis using a matched population from Taif, western Saudi Arabia. The burden of protozoan infections, associated symptoms, and potential risk factors were also sought in this study.

## 2. Materials and Methods

### 2.1. Study Site and Population

Two hundred and four patients comprising 139 with CRF and 64 without CRF from those patients attending the outpatient clinics at King Abdulaziz Specialized Hospital, Taif, Saudi Arabia, seeking treatment of their diarrheal episodes, were invited to participate in the study.

### 2.2. Exclusion Criteria

An episode of diarrhea was defined as ≥3 loose bowel movements over a day prior to interviewing. Individuals with history of diabetes mellitus, malignancy, autoimmune disease, or any other chronic diseases were excluded from the study. Patients with history of taking antiparasitic, antidiarrheal, or immunosuppressive medications, 2 weeks prior to meeting, were also excluded from participation in the study. Following these exclusion criteria, 50 patients with CRF, described as cases, and 50 patients without CRF, named as controls, were allocated in the current study.

### 2.3. Ethical Consideration

All participants were informed of the purpose of our study and signed consent forms authorizing their voluntary participation. The regional ethics committee approved data collection, clinical samples collection, and analysis of the study.

### 2.4. Interviews and Questionnaire

On enrollments, face-to-face interviews were conducted using a structured questionnaire for the basic demographic data in terms of age, sex, and residence. Questions about drinking water resources, history of travel to foreign countries, and/or exposure to animals during the three-week period preceding diarrhea were also included. Moreover, some clinical data concerning the number of bowels/day, duration of diarrhea, accompanying nausea/vomiting, mucous or blood in feces, abdominal pain, bloating/flatulence, general fatigue, and loss of weight were also sought for in the questionnaire.

### 2.5. Fecal Samples Collection

A total of 100 fecal samples, one sample from each participant, were collected between June and December 2014. Fresh samples were obtained in clean screw-capped cups. Feces were transported to the Microbiology Laboratory at College of Applied Medical Sciences, Taif University, within 2-3 hours after collection. Samples were appropriately coded and immediately stored at 4°C till the time of parasitological examination.

### 2.6. Parasitological Examination

Fresh feces were examined for consistency and presence of mucous or blood. Direct wet mount smears were microscopically examined for parasites ova, cysts, oocysts, and/or larvae, as previously described [14]. Formol-ether fecal concentrates of each sample were individually smeared and stained with native iodine, trichrome, modified trichrome, and modified Ziehl-Neelsen stains for identification of* Blastocystis* spp.,* Microsporidium* spp., and intestinal coccidian protozoa, respectively. Staining and examination techniques were carried out according to Garcia, 2009 [15].

Fresh fecal specimens were also examined for* G*.* duodenalis* with RIDA Quick Giardia (R-Biopharm, Darmstadt, Germany),* Cryptosporidium* spp. with RIDA Quick Cryptosporidium (R-Biopharm, Darmstadt, Germany), and for* E. histolytica* with E. histolytica II Test (TechLab, Blacksburg, Virginia, USA) commercial kits. Immunoassays were performed following the corresponding manufacturer's protocol.

### 2.7. Statistical Methods

Statistical package for social science (SPSS) program, version 16 for windows, was used for data entry and data analysis. Summaries with descriptive statistics were generated and the data was further statistically analyzed according to the objectives of the study. Appropriate statistical tests (parametric or nonparametric tests) were used according to the type of data whether qualitative or quantitative. *P* value ≤ 0.05 was considered significant.

## 3. Results

### 3.1. Demographic Characteristics

Among 100 participants, 67 were males (mean age of 54.4 ± 12.8) and 33 were females (mean age of 51.9 ± 14.3). Eighty-three participants were urban, while the remaining 13 participants were from remote rural areas. Forty-seven patients had travel history to one tropical country 1–3 months before their diarrheal episodes. Twenty-six participants had contact with one or more domestic animal 3-4 weeks prior to their diarrheal episodes. Treated bottled water was the main drinking water resource for 56 participants, followed by desalted sea water for 31 participants and the underground well water for 13 participants.

### 3.2. Parasitic Infections

**Figure 1:**
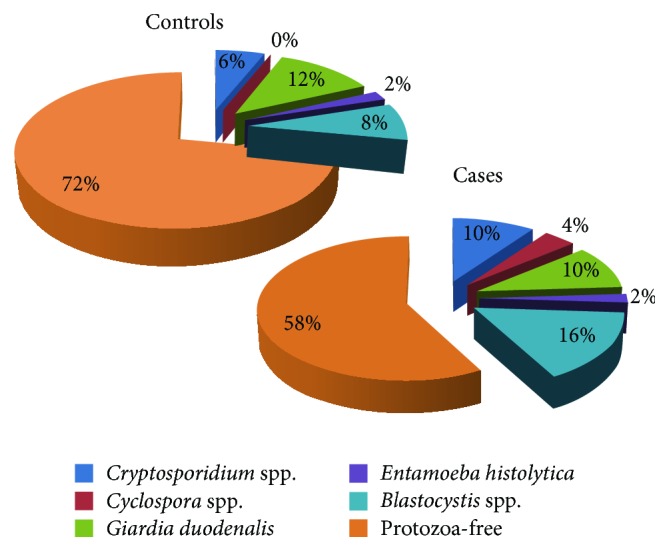
Parasitological tests results for 50 cases and 50 controls. Percentages of protozoan infection were written inside or outside color-matched pie charts slices for comparison.

Intestinal protozoa were detected in 21 (42%) cases and 14 (28%) controls, with an overall prevalence rate of 35%. No significant differences were statistically observed between both groups (*P* = 0.14).* Blastocystis* spp. were the most frequently identified parasite (12/100; 12%) followed by* G. duodenalis* (11/100; 11%) and* Cryptosporidium* spp. (8/100; 8%).* Blastocystis* spp.,* Cryptosporidium* spp.,* G. duodenalis*,* C. cayetanensis,* and* E. histolytica* were identified in cases at infection rates of 16% (8/50), 10% (5/50), 10% (5/50), 4% (2/50), and 2% (1/50), respectively. In the control group,* Blastocystis* spp.,* Cryptosporidium* spp.,* G. duodenalis,* and* E. histolytica* were diagnosed with prevalence rates of 8% (4/50), 6% (3/50), 12% (6/50), and 2% (1/50), respectively (Figure 1). None of these infections showed significant statistical difference between the two groups.

**Figure 2:**
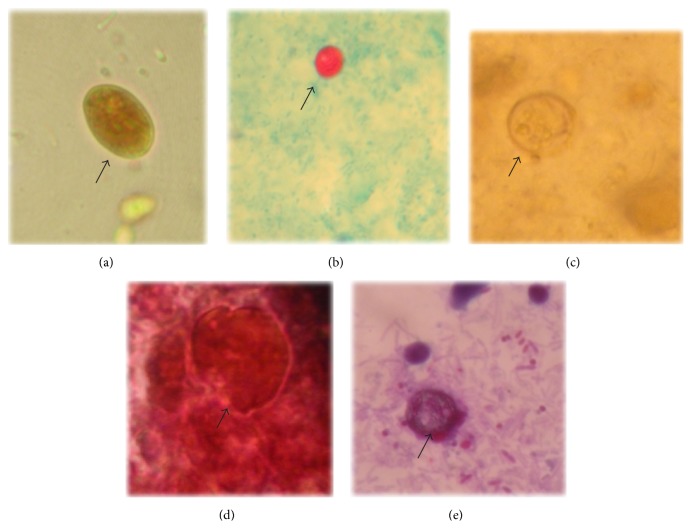
Merged microscopic pictures for wet mounts smears of formol-ether fecal concentrates showing the recognized enteric protozoa. (a)* Giardia duodenalis* cyst (iodine stain, 400x magnification), (b)* Cryptosporidium* spp. oocyst (modified Ziehl-Neelsen stain, 400x magnification), (c)* Entamoeba histolytica/Entamoeba dispar* cyst (iodine stain, 400x magnification), (d)* Blastocystis* spp. vacular form (trichrome stain, 1000x magnification), and (e)* Cyclospora* spp. oocyst (mZN stain, 400x magnification).

Concomitant parasitic infections were described in 5 cases (5/100; 5%) and were absent in the control group.* Cryptosporidium* spp. were concurrently identified with* G. duodenalis* in a case and with* C. cayetanensis* in another.* Blastocystis* spp. were concomitantly detected with* E. histolytica* in a case and with* G. duodenalis* in two cases. No helminths infection was observed for either cases or the control group. Neither* C. belli* nor Microsporidia spp. were detected in the study's participants. Importantly, 2 participants, one from each group, diagnosed as positives for* E. histolytica*/*E. dispar* by microscopy, were proved as* E. histolytica *positives by the ELISA test. Equally important, identification of protozoan cysts/oocysts in patients feces (Figure 2) was confirmed by the corresponding immunoassay.

### 3.3. Parasitism and Patients' Demographic Characteristics

Concerning the parasitized cases (*n* = 21), 14 (66.7%) were males and 7 (33.3%) were females. Infections were more frequent in cases from urban areas (16/21; 76.2%) than those residing rural areas (5/21; 23.8%). Eleven cases (11/21; 52.4%) had travel history and 10 (10/21; 47.6%) cases had not. Among the parasitized controls (*n* = 14), intestinal protozoa were identified more in males (9/14; 64.3%) than in females (5/14; 35.7%), in persons from urban areas (9/14; 64.3%) than those residing rural areas (5/14; 35.7%), and in participants with travel history (7/14; 50%) than those without (7/14; 50%). No significant differences were statistically observed for any of these variables between the parasitized cases and controls (Table 1).

### 3.4. Parasitism and Patients' Clinical Characteristics

Acute and transient diarrhea was reported in 71% (15/21) of the parasitized cases and by all parasitized controls (14/14; 100%), with no significant differences observed (*P* = 0.06) between both groups. Diarrhea continued for a duration of more than 2 weeks only in 28.6% (6/21) of protozoa-positive cases. Persistent diarrhea was reported in two* G. duodenalis* positive cases, two* Cryptosporidium* spp. positive cases, one* Blastocystis* spp. positive case, and one* C. cayetanensis* positive case. Mucous and blood were seen in feces of two cases (9.5%; 2/21) and one control (7.1%; 1/14). One of these two cases was positive for* E. histolytica* and* Blastocystis* spp., while the other was positive for* Blastocystis* spp., as a sole infection. The only patient in the control group who reported mucous and blood in his feces was* E. histolytica* positive. Bloating/flatulence, abdominal pain, general fatigue, and nausea/vomiting were the common symptoms found associated with the diarrhea (Table 2).

Diarrhea associated with abdominal pain and fatigue sensation was reported by all the parasitized cases. Diarrhea associated with nausea/vomiting, bloating/flatulence sensation, and losing some weight was reported by ≈57% (12/21), ≈76% (16/21), and 9.5% (2/21) of the parasitized cases, respectively. Among the parasitized controls (*n* = 14), diarrhea accompanied with symptoms of abdominal pain, nausea/vomiting, bloating/flatulence sensation, feeling fatigued, and losing weight was practiced by ≈79% (11/14), 93% (13/14), ≈71% (10/14), and 100% (14/14) of patients, respectively. Significant differences were found between parasitized cases and parasitized controls regarding the association of their diarrhea with abdominal pain (*P* = 0.05) and with nausea/vomiting (*P* = 0.02). No significant difference between the parasitized cases and controls was observed for other symptoms.

### 3.5. *Blastocystis* Species Associated Diarrhea and Patients' Clinical Characteristics

Diarrhea was often mild (87.5% of cases versus 100% of controls) and nondysenteric (87.5% of cases versus 100% of controls). The commonly associated symptoms were abdominal pain, fatigue (100% of cases and controls), nausea/vomiting (87.5% of cases versus 100% of controls), flatulence/bloating (75% of cases versus 25% of controls), and losing weight (12.5% of cases versus 0% of controls). No significant difference was found between the parasitized cases and controls (Table 3).

### 3.6. *Cryptosporidium* Species Associated Diarrhea and Patients' Clinical Characteristics

*Cryptosporidium* species associated diarrhea was often mild (60% of cases versus 100% of controls) and nondysenteric in all parasitized patients. The commonly reported associated symptoms were fatigue (100% of cases and controls), abdominal pain (100% of cases versus 33.3% of controls), nausea/vomiting (60% of cases versus 100% of controls), flatulence/bloating (80% of cases versus 100% of controls), and losing weight (12.5% of cases and controls). Statistically, no significant difference was described for any of these symptoms between the parasitized patients in both groups (Table 4).

### 3.7. *Giardia duodenalis* Associated Diarrhea and Patients' Clinical Characteristics

As described in Table 5,* G. duodenalis* associated diarrhea was frequently mild (60% of cases versus 100% of controls) and nondysenteric (100% of cases and 83.3% of controls). The common associated symptoms were fatigue (100% of cases and controls), abdominal pain (100% of cases versus 83.3% of controls), nausea/vomiting (80% of cases versus 83.3% of controls), flatulence/bloating complaints (100% of cases versus 83.3% of controls), and losing weight (00% of cases versus 9% of controls). None of these symptoms showed significant statistical differences between the parasitized cases and controls.

## 4. Discussion

In the study's population, protozoan infections associated with diarrhea were common (35%). Infections were identified in considerable number of patients in the diarrheal control group. This is not surprising for this population where several protozoan infections predominate [12]. Intestinal protozoa were identified more in cases than in control group, reflecting the high association between the isolated protozoa and diarrhea in patient with CRF. In agreement with this finding, in one case-control study carried out in Turkey, protozoan infections have been described in ≈44% (62/142) of cases and ≈13% (19/150) of controls [16]. In another study executed in Brazil, Kulik et al. have reported these infections in ≈45% (33/86) of cases and in ≈26% (36/146) of controls [17]. Contrary to our findings, Gil et al. have reported more protozoan infections (61%) in controls than in cases from Brazil (≈52%) [18]. It is far important to announce that the three aforementioned studies were community based. According to the literature, infections with intestinal protozoan infections are commonly asymptomatic [1]. Therefore, high proportions of asymptomatic infections are expected in these studies; on the contrary, in hospital or physician-based study, like our study, where the control group was selected from those patients suffering from diarrhea, asymptomatic protozoan infection was misdiagnosed. This may put an explanation for the high frequency of protozoan infections in the above studies, especially in the control groups compared to our study. Perhaps, variation of the geographical distribution of protozoa, the socioeconomic status of the target population, the demographic characters of participants, and the adopted methodology may be additional explanations for prevalence variations of these protozoan infections among studies.

It has been argued that CRF predispose patients to helminths [11] but this was not the case in our study where no helminths were detected in all participants. Most of our study's participants were urban, middle aged, and using treated bottled water for drinking. These demographic characters may give reasons for the absence of worms' infections that have been broadly recognized by their intimate connection with poverty, remote rural areas, and young ages [19]. Another important reason to be considered is that patients with CRF usually change their health behavior and receive much more health care attention than their peers in populations.

*Blastocystis* spp. were the most frequently isolated protozoa in the present study. These protozoa were detected more in cases than in controls, consistent with earlier studies [16, 17]. Higher prevalence rates (≈24%; 34/142 in cases versus 10%; 16/150 in the controls) of* Blastocystis* spp. infections have been described in the Turkish study [16]. In the Brazilian study, infections have been described in cases (21%; 18/86) only [17]. Inconsistent with our finding,* Blastocystis* spp. have been identified more in controls (≈42%; 36/86) than in cases (24.5%; 27/110), according to Gil et al. [18]. In a cross-sectional Iranian study,* Blastocystis* spp., with prevalence rate of 13.6% (12/88), have been reported in dialysis patients [20]. Lower prevalence rates (0.3–14%) have been previously reported for this intestinal protozoan in Saudi normal populations [21–25].

Considering the opportunistic enteric protozoa, neither* C. belli* nor Microsporidia spp. were identified in our study. Except for a recently published case report for* C. belli* infection overwhelming a patient with eosinophilic gastroenteritis [26], little has been published for the frequency of these protozoa in Saudi Arabia. Low frequency of* Microsporidium* spp. infections (2%; 3/142) has been reported in Turkish patients with CRF [16]. High prevalence rate of* Cryptosporidium* spp. was revealed in the present study. While higher rates (11–35%) have been described in earlier studies [27–29], lower estimates (2–8%) have been reported in others [16, 30].* Cryptosporidium* spp. infection has been stated with prevalence rate of 2.7–8.1% in Saudi general populations [23, 25]. Identification of* C. cayetanensis* in two patients with CRF was interesting. To the best of our knowledge, this protozoan has never been reported in patients with CRF. In Saudi Arabia,* C. cayetanensis* has been rarely detected even in immunocompromised patients [23].

*Giardia duodenalis* was detected in considerable number of the study's participants. Infections were identified more in the control group, compatible with an earlier study [18] and incompatible with another [16].* Giardia duodenalis* has been commonly detected in Saudi communities. Infection rates of 3–18% have been reported [12, 22–26]. Moreover,* E. histolytica*, the tissue-invasive intestinal protozoan, was rarely determined among cases in this study, consistent with a previous study [17] and inconsistent with other [31].* E. histolytica* infection has been recognized as a public health threat in Saudi Arabia. Prevalence rates of 6–35% have been described in Saudi Arabia [13, 21–24]. Interestingly, in a study carried out in Saudi population, an infection rate of ≈60% has been described [25]. In the aforementioned studies, microscopy has been used as a sole technique for diagnosis of amoebiasis. The low sensitivity of microscopy and its incompetence to determine the morphologically identical species may put an explanation for these exaggerated prevalence rates.

Diarrhea was often acute and transient in most of the parasitized patients. Persistent diarrhea was reported by few parasitized cases, consistent with previous report [32]. Persistent diarrhea was described with all the recognized protozoa except* E. histolytica*. Acute dysenteric diarrhea was described in six cases, two of them were found parasitized with* Blastocystis* spp. concomitantly with* G. duodenalis* in one patient and with* E. histolytica* in the other. In addition, acute dysenteric diarrhea was observed in three controls, one of them was found parasitized with* E. histolytica*. In the presence of coinfection, it is very challenging to attribute patients' symptoms to one protozoan and neglect the other [33]. Perhaps, one may attribute dysentery to* E. histolytica* being a well-known tissue-invasive parasite [31]. One else may relate the dysenteric diarrhea to* Blastocystis* infection relying on a previous research [34, 35]. Any other dysenteric cases that have not been supported by the literature require further investigations.

This is the first study comparing the frequency of intestinal protozoa-associated diarrhea in CRF patients and nonrenal controls from Taif, Saudi Arabia. In this study, special considerations were given to avoid well-known potential confounders while enrolling patients and subsequent selection bias. Another strength of our study was the inclusion of a number of immunoassays in the study's methodology to avoid the low sensitivity of microscopy-based methods in diagnosis of certain parasites such as* E. histolytica*. Nevertheless, our study was not free from the limitations. Low number of cases was enrolled in this study due to the strict selection criteria adopted. We could not rely on the recent immune status of participants based on doing laboratory tests because many participants were reluctant to give blood samples. Lastly, we did not include tests for intestinal bacteria and viruses that could be concomitantly present with protozoan infection(s), due to time and cost limits of our study. All these limitations plus performing longitudinal study have to be considered in the near future research.

In conclusion, high frequency of intestinal protozoan infections was described in the study's population. Protozoa were detected more in patients with CRF than in diarrheal controls. Further studies are required before attributing diarrhea in patients with CRF to a protozoan detected in patients' feces. Until these studies, we advise screening all diarrheal patients for intestinal protozoa in the study's population, with greater concern that should be given to patients with CRF.

## Figures and Tables

**Table 1:** The demographic characteristics variations among the parasitized and nonparasitized diarrheal cases and controls.

Demographic character	Protozoa-positive					Protozoa-negative				
	Cases		Controls		*P* value	Cases		Controls		*P* value
	*n*	%	*n*	%		*n*	%	*n*	%	
Age										
Mean ± SD	54.4 ± 12.8		48.8 ± 12.6		0.2 (NS)	48.6 ± 13.2		51.9 ± 14.3		0.33 (NS)
Gender										
Male	14	66.7%	9	64.3%	0.88 (NS)	21	72.4%	23	63.9%	0.46 (NS)
Female	7	33.3%	5	35.7%		8	27.6%	13	36.1%	
Residence										
Urban	16	76.2%	9	64.3%	0.47 (NS)	27	93.1%	31	86.1%	0.44 (NS)
Rural	5	23.8%	5	35.7%		2	6.9%	5	13.9%	
Travel history										
Yes	11	52.4%	7	50%	0.89 (NS)	14	48.3%	15	41.7%	0.59 (NS)
No	10	47.6%	7	50%		15	51.7%	21	58.3%	
Animal contact										
Yes	7	33.3%	6	42.9%	0.56 (NS)	7	24.1%	6	16.7%	0.45 (NS)
No	14	66.7%	8	57.1%		22	75.9%	30	83.3%	
Drinking water										
Bottled	11	52.4%	6	42.9%	0.83 (NS)	19	65.5%	20	55.6%	0.007 (Sig)
Desalted	8	38.1%	6	42.9%		3	10.3%	14	38.9%	
Well	2	9.5%	2	14.3%		7	24.1%	2	5.6%	
Total	21	100%	14	100%		29	100%	36	100%	

SD: standard deviation, *n*: number, NS: nonsignificant, and Sig: significant. *P* < 0.005.

**Table 2:** The clinical characteristics variations among the parasitized and non-parasitized diarrheal cases and controls.

Clinical character	Protozoa-positive					Protozoa-negative				
	Cases		Controls		*P* value	Cases		Controls		*P* value
	*n*	%	*n*	%		*n*	%	*n*	%	
Diarrhea										
<14 days	15	71.4%	14	100%	0.06 (NS)	25	86.2%	36	100%	0.03 (Sig)
>14 days	6	28.6%	0	0%		4	13.8%	0	0%	
Mucous/blood										
Yes	2	9.5%	1	7.1%	1.0 (NS)	4	13.8%	2	5.6%	0.39 (NS)
No	19	90.5%	13	92.9%		25	86.2%	34	94.4%	
Abdominal pain										
Yes	21	100%	11	78.6%	0.05 (Sig)	29	100%	28	77.8%	0.007 (Sig)
No	0	0%	3	21.4%		0	0%	8	22.2%	
Nausea/vomiting										
Yes	12	57.1%	13	92.9%	0.02 (Sig)	2	6.9%	4	11.1%	0.68 (NS)
No	9	42.9%	1	7.1%		27	93.1%	32	88.9%	
Bloating/flatulence										
Yes	16	76.2%	10	71.4%	1.0 (NS)	18	62.1%	6	16.7%	0.001 (HS)
No	5	23.8%	4	28.6%		11	37.9%	30	83.3%	
Fatigue										
Yes	21	100%	14	100%	—	29	100%	4	11.1%	0.001 (HS)
No	0	0%	0	0%		0	0%	32	88.9%	
Loss of weight										
Yes	2	9.5%	2	14.3%	1.0 (NS)	4	13.8%	4	11.1%	0.74 (NS)
No	19	90.5%	12	85.7%		25	85.2%	32	88.9%	
Total	21	100%	14	100%		29	100%	36	100%	

*n*: number, NS: nonsignificant, Sig: significant, and HS: highly significant. *P* < 0.005.

**Table 3:** The clinical characteristics variations among *Blastocystis* species positive/negative diarrheal cases and controls.

Clinical character	*Blastocystis* species-positive					*Blastocystis *species-negative				
	Cases		Controls		*P* value	Cases		Controls		*P* value
	*n*	%	*n*	%		*n*	%	*n*	%	
Diarrhea										
<14 days	7	87.5%	4	100%	1.0 (NS)	33	78.6%	46	100%	0.001 (Sig)
>14 days	1	12.5%	0	0%		9	21.4%	0	0%	
Mucous/blood										
Yes	1	12.5%	0	0%	1.0 (NS)	5	11.9%	3	6.5%	0.47 (NS)
No	7	87.5%	4	100%		37	88.1%	43	93.5%	
Abdominal pain										
Yes	8	100%	4	100%	—	42	100%	35	76.1%	0.001 (Sig)
No	0	0%	0	0%		0	0%	11	23.9%	
Nausea/vomiting										
Yes	7	87.5%	4	100%	1.0 (NS)	7	16.7%	13	28.3%	0.2 (NS)
No	1	12.5%	0	0%		35	83.3%	33	71.7%	
Bloating/flatulence										
Yes	6	75%	1	25%	0.22 (NS)	28	66.7%	15	32.6%	0.003 (Sig)
No	2	25%	3	75%		14	33.3%	34	67.4%	
Fatigue										
Yes	8	100%	4	100%	—	42	100%	14	30.4%	0.00 (HS)
No	0	0%	0	0%		0	0%	32	69.6%	
Loss of weight										
Yes	1	12.5%	0	0%	1.0 (NS)	5	11.9%	6	13%	1.0 (NS)
No	7	87.5%	4	100%		37	88.1%	40	87%	
Total	8	100%	4	100%		42	100%	46	100%	

*n*: number, NS: nonsignificant, Sig: significant, and HS: highly significant. *P* < 0.005.

**Table 4:** The clinical characteristics variations among *Cryptosporidium* species positive/negative diarrheal cases and controls.

Clinical character	*Cryptosporidium* species-positive					*Cryptosporidium* species-negative				
	Cases		Controls		*P* value	Cases		Controls		*P* value
	*n*	%	*n*	%		*n*	%	*n*	%	
Diarrhea										
<14 days	3	60%	3	100%	0.46 (NS)	37	82.2%	47	100%	0.002 (Sig)
>14 days	2	40%	0	0%		8	17.8%	0	0%	
Mucous/blood										
Yes	0	0%	0	0%	—	6	13.3%	3	6.4%	0.31 (NS)
No	5	100%	3	100%		39	86.7%	44	93.6%	
Abdominal pain										
Yes	5	100%	1	33.3%	0.10 (NS)	45	100%	38	80.9%	0.003 (Sig)
No	0	0%	2	66.7%		0	0%	9	19.1%	
Nausea/vomiting										
Yes	3	60%	3	100%	0.46 (NS)	11	24.4%	14	29.8%	0.64 (NS)
No	2	40%	0	0%		34	75.6%	33	70.2%	
Bloating/flatulence										
Yes	4	80%	3	100%	1.0 (NS)	30	66.7%	13	27.7%	0.000 (HS)
No	1	20%	0	0%		15	33.3%	34	72.3%	
Fatigue										
Yes	5	100%	3	100%	—	45	100%	15	31.9%	0.000 (HS)
No	0	0%	0	0%		0	0%	32	68.1%	
Loss of weight										
Yes	1	20%	1	33.3%	1.0 (NS)	5	11.1%	5	10.6%	1.0 (NS)
No	4	80%	2	66.7%		40	88.9%	42	89.4%	
Total	5	100%	3	100%		45	100%	47	100%	

*n*: number, NS: nonsignificant, Sig: significant, and HS: highly significant. *P* < 0.005.

**Table 5:** The clinical characteristics variations among *Giardia  duodenalis* positive/negative diarrheal cases and controls.

Clinical characters	*Giardia duodenalis*-positive					*Giardia duodenalis*-negative				
	Cases		Controls		*P* value	Cases		Controls		*P* value
	*n*	%	*n*	%		*n*	%	*n*	%	
Diarrhea										
<14 days	3	60%	6	100%	0.18 (NS)	37	82.2%	44	100%	0.006 (Sig)
>14 days	2	40%	0	0%		8	17.8%	0	0%	
Mucous/blood										
Yes	0	0%	1	16.7%	1.0 (NS)	6	13.3%	2	4.5%	0.26 (NS)
No	5	100%	5	83.3%		39	86.7%	42	95.5%	
Abdominal pain										
Yes	5	100%	5	83.3%	1.0 (NS)	45	100%	34	77.3%	0.00 (HS)
No	0	0%	1	16.7%		0	0%	10	22.7%	
Nausea/vomiting										
Yes	4	80%	5	83.3%	1.0 (NS)	10	22.2%	12	27.3%	0.62 (NS)
No	1	20%	1	16.7%		35	77.8%	32	72.7%	
Bloating/flatulence										
Yes	5	100%	5	83.3%	1.0 (NS)	29	64.4%	11	25%	0.000 (HS)
No	0	0%	1	16.7%		16	35.6%	33	75%	
Fatigue										
Yes	5	100%	6	100%	—	45	100%	12	27.3%	0.000 (HS)
No	0	0%	0	0%		0	0%	32	72.7%	
Loss of weight										
Yes	0	0%	1	16.7%	1.0 (NS)	6	13.3%	5	11.4%	1.0 (NS)
No	5	100%	5	83.3%		39	86.7%	39	88.6%	
Total	5	100%	6	100%		45	100%	44	100%	

*n*: number, NS: nonsignificant, Sig: significant, and HS: highly significant. *P* < 0.005.
